# Dr. J. F. Krug

**Published:** 1907-02

**Authors:** 


### OBITUARY.

---

Dr. J. F. Krug, a graduate of the Indiana Medical College in 1879, died of pneumonia on December 31, 1906, at his home, 744 Filmore avenue, Buffalo. Dr. Krug was born in Mecklenburg-Schwerin, Germany, and was educated in public and private schools. He came to this country at the close of the Franco-Prussian war and settled in Buffalo where he did art work. Later he went to Indianapolis and studied medicine. After graduation he returned to Buffalo and began practice. He was a member of the medical society of the county of Erie. The funeral was held January 3, 1907.

